# *C. elegans* LRP-2 functions in vulval precursor cell polarity

**DOI:** 10.17912/micropub.biology.000152

**Published:** 2019-08-27

**Authors:** Paul J Minor, Paul W Sternberg

**Affiliations:** 1 Division of Biology and Biological Engineering, Caltech, Pasadena, CA 91125; 2 Department of Biology, Hopkins Marine Station of Stanford University, Pacific Grove, CA 93950

**Table 1. LRP-2 functions in ground polarity:**
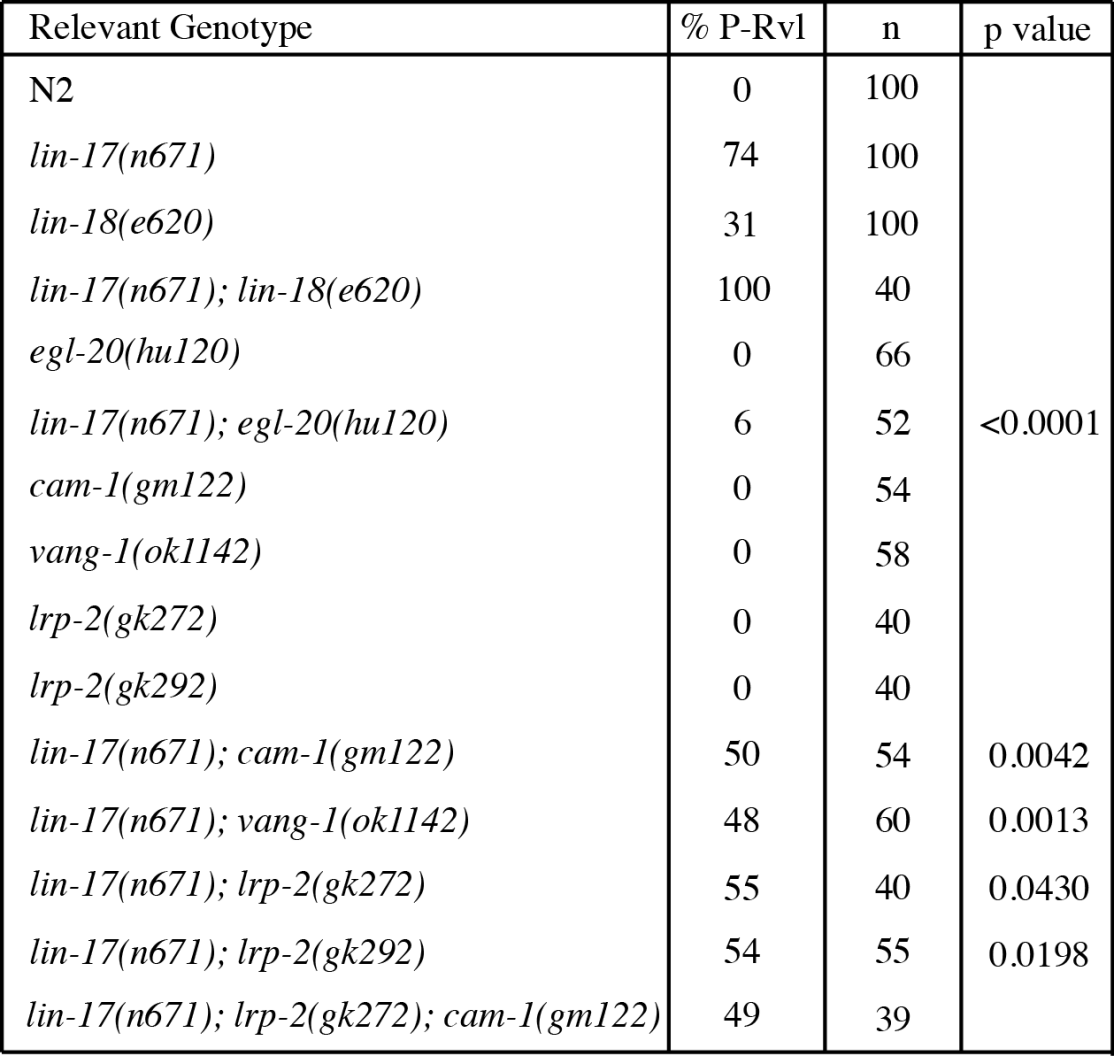
P values in comparison to *lin-17(n671)* single mutants using Fisher’s Exact Test.

## Description

The *C. elegans* vulva is formed from divisions of three vulval precursor cells (VPCs) – P5.p, P6.p, and P7.p – arranged along the anteroposterior axis in the ventral epithelium (Sulston and Horvitz, 1977). Previous analyses show the orientation of P5.p and P7.p descendants is determined by the interaction of multiple Wnt signals. Specifically, in the absence of all Wnts, the VPCs display a randomized orientation, which is likely the default (Green *et al*., 2008; Minor *et al*. 2013). Two separate Wnts from the anchor cell, LIN-44 and MOM-2 acting through receptors LIN-17/Frizzled and LIN-18/Ryk, respectively, regulate P7.p orientation (Ferguson *et al*., 1987; Sternberg and Horvitz, 1988; Sawa *et al*., 1996; Inoue *et al*., 2004; Gleason *et al*., 2006). In the absence of these signals the orientation of the progeny of P7.p mimic those of P5.p and face toward the posterior of the worm, a phenotype referred to as posterior-reversed vulval lineage (P-Rvl). This posterior orientation is dependent on the instructive signal EGL-20, a Wnt expressed in the tail acting through CAM-1/ROR and VANG-1/Van Gogh, and is referred to as “ground polarity” (Green *et al*., 2008).

Here we examine the role of a low-density lipoprotein receptor, *lrp-2*, and its role in controlling the orientation of P7.p daughter cells. To investigate this interaction double mutants were constructed with both alleles of *lrp-2* and *lin-17(n671)* (Table 1). Much like *cam-1(gm122)* and *vang-1(ok1142)*, both alleles of *lrp-2* suppress the *lin-17(n671)* phenotype from 74 to approximately 50% P-Rvl leading us to hypothesize that *lrp-2* functions in the same pathway as *cam-1* and *vang-1.* Furthering this hypothesis we have shown that, like *cam-1* and *vang-1, lrp-*2 controls the localization of SYS-1/b-catenin (Minor and Sternberg, 2019). To ensure that this phenotype was a result of loss of *lrp-2* function as opposed to background effects we injected a fosmid (WRM0617cA02) containing the full-length sequence of *lrp-2* and found that it does rescue the double mutant phenotype of *lin-17(n671); lrp-2(gk272)* from 55 to 73%. In order to better test this hypothesis a triple mutant was constructed between *lin-17(n671), lrp-2(gk272)*, and *cam-1(gm122)* (Table 1). This triple mutant displays the same P-Rvl penetrance as both the *lin-17(n671); lrp-2(gk272)* and *lin-17(n671); cam-1(gm122)* double mutants confirming that *lrp-2* functions in the same pathway as *cam-1.*

## Methods

Strains were constructed by standard procedures (Ferguson and Horvitz, 1985).

## Reagents

**Strains:**

N2

**MT1306**: *lin-17(n671)* (Ferguson and Horvitz, 1985)

**PS4818**: *lin-18(e620)*

**PS3976:** *lin-17(n671)/ hT2 [qIs48 (myo-2::gfp etc.)]; +/ hT2 [qIs48]; lin-18(e620)*

**PS5401**: *egl-20(hu120)*

**PS5530**: *lin-17(n671); egl-20(hu120)*

**NG2615**: *cam-1(gm122)*

**RB1125**: *vang-1(ok1142)*

**VC543**: *lrp-2(gk272)*

**VC621**: *lrp-2(gk292)*

**PS4809**: *lin-17(n671); cam-1(gm122)*

**PS5651**: *lin-17(n671); vang-1(ok1142)*

*lin-17(n671); lin-18(e620)* animal awere segregated from **PS3976.**

The *lin-17(n671); lrp-2(gk292)* double mutant was constructed by crossing *lrp-2(gk292)* males with strain **MT1488**: *lin-17(n671); unc-13(e1091)* hermaphrodites.

The *lin-17(n671); lrp-2(gk272)* double mutant constructed by crossing *lrp-2(gk272)* males with strain **MT1488**: *lin-17(n671); unc-13(e1091)* hermaphrodites.

*lin-17(n671); lrp-2(gk272); cam-1(gm122)* Triple mutant constructed by crossing *lin-17(n671); lrp-2(gk272)* hermaphrodites with *cam-1(gm122)* and sequencing for homozygous triples.

**PS6581**: *qIs95[pSYS-1::VENUS::SYS-1 with pttx-3::dsRed as coninjection marker]*;

**PS6576:** *lin-18(e620) sem-5(n1779)*

**PS6580**: *qIs95; sem-5(n1779)*

**PS6579**: *Pegl-17::CWN-1::GFP (unnamed construct) in cwn-1(ok546); lin-18(e620)*
